# Adoption of video consultations during the COVID-19 pandemic

**DOI:** 10.1016/j.invent.2023.100602

**Published:** 2023-01-20

**Authors:** Filipe Viana Pereira, Jorge Tavares, Tiago Oliveira

**Affiliations:** NOVA Information Management School (NOVA IMS), Universidade Nova de Lisboa, Campus de Campolide, 1070-312 Lisboa, Portugal

**Keywords:** Video consultations, Technology adoption, Telemedicine, Patient, Health, DOI, UTAUT, HBM, CFIP

## Abstract

**Background:**

Video consultations have the potential to play a significant role for the future of healthcare by solving some of the imminently arising healthcare challenges, as pointed by the European Commission in Europe and the National Academy of Medicine in the United States of America. This technology can improve quality, efficiency, and enhance access to healthcare.

**Objective:**

The aim of this study is to explore and understand individual video consultations acceptance drivers.

**Methods:**

An extended technology acceptance model was created based on the diffusion of innovation theory (DOI), unified theory of acceptance and use of technology (UTAUT), health belief model (HBM), and concerns for information privacy framework (CFIP). 346 valid responses were collected through an online questionnaire, and the partial least squares (PLS) modeling approach was used to test the model.

**Results:**

The model explained 77.6 % (R2) of the variance on intention to use, and 71.4 % (R2) of the variance in attitude. The predictors of intention to use are attitude (beta = 0.504, *p*-value<0.001), performance expectancy (beta = 0.196, *p*-value = 0.002), and COVID-19 (beta = 0.151, *p*-value<0.001). The predictors of attitude are performance expectancy (beta = 0.643, p-value>0.001), effort expectancy (beta = 0.138, p-value = 0.001), and COVID-19 (beta = 0.170, p-value<0.001).

**Conclusions:**

This research model highlights the importance of creating extended acceptance models to capture the specificities of each technology in healthcare. The model created helps to understand the most important drivers of video consultation acceptance, highlighting the importance of the COVID-19 pandemic and perceived health risks.

## Highlights

• Video consultations were a solution to the difficult access to healthcare services during COVID-19, and the learnings can be used to further extend the use of video consultations.
• An extend technology acceptance model was created based on the DOI, UTAUT, HBM, and CFIP.
• The most significant constructs were performance expectancy, effort expectancy, compatibility, the COVID-19 pandemic, and perceived health risk.
• Managerial implications are provided to improve the acceptance of video consultations.

## 1 Introduction

Telemedicine is heralded as the future of health care in several parts of the world. The European Commission and the National Academy of Medicine in the United States of America, highlighted telemedicine's importance for the future (Communication From the Comission to the European Parliament, the Council, the European Economic and Social Committee and the Committee of the Regions: EHealth Action Plan, 2012) (Dzau et al., 2020). According to the World Health Organisation, telemedicine is the “delivery of healthcare services, where patients and providers are separated by distance, using information communication technologies for the exchange of information for diagnosis, treatment, and prevention of diseases and injuries, research and evaluation, and the continuing education of health professionals” (Ho et al., 2009). Video consultations, a specific type of telemedicine, can be defined as a two-way audio-visual synchronous conference between a patient and a clinician (Donelan et al., 2019).

During the COVID-19 pandemic, there was an increase in the use of telemedicine for urgent and non-urgent care visits (Mann et al., 2020). According to Jiménez Rodriguez et al.'s article “Increase in video consultations during the COVID-19 pandemic”, a survey found that 96.2 % of healthcare professionals considered video-conference consultations an adequate option for providing health care (Jiménez-Rodríguez et al., 2020). On the patient perceptions side, some state that video consultations would be their preferred method of visit, while for others it would be a supplementary way to consult their doctor, while highlighting it is perceived as more patient centric (Thiyagarajan et al., 2020).Patient satisfaction with video consultations ranges from 58 % to 90 % according to recently published studies (Anderson et al., 2021; Parker and Chia, 2021).

This study aims to understand what drives consumers to use video consultations and whether the COVID-19 pandemic influenced the adoption of video consultations.

### 1.1 Theoretical background

Several theories show potential to support the understanding of video consultations adoption, as shown in Table 1**.** However, still specific studies regarding this technology are lacking.

**Table 1:** Patient adoption models relevant for video consultations.

Theory	Relevance for video consultation	Reference
UTAUT	The Unified Theory of Acceptance and Use of Technology (UTAUT) is one of the most used adoption theories in the field of technology adoption. It has also been used to study video consultation with good results. Four main constructs are used in this theory, all were used in this this study: • Performance Expectancy (PE): The expectation that this technology will help the user to manage more efficiently their health. • Effort Expectancy (EE): The concept that the technology should be easy to use. • Social Influence (SI): The degree to which an individual perceives that others important to him or her believe he or she should use the technology. • Facilitating Conditions (FC): Meaning that the necessary resources are available to support the use of the technology.	Kim and Park, 2012; Rho et al., 2015; Alam et al., 2020
HBM	Video consultation are a healthcare patient – centric technology. The use of the Health Belief Model (HBM), a specific model connected with healthcare showed good results with technologies that support video consultation, like mHealth and patient portals. Perceived Health Risk (PHR), the idea that the individual's perception of their health condition drives the behaviour towards corrective health actions showed good results when used as an extension to other models like UTAUT. PHR was used as a second order construct in this study	Tavares and Oliveira, 2016; Ahadzadeh et al., 2015
DOI	The use of video consultation with new technologies, like mobile devices, mHealth and Patient Portals, together with a recent increased usage growth, makes it suitable to be studied with the Diffusion of Innovation Theory (DOI). Recent studies showed promising results with the use of DOI. The construct Complexity from DOI corresponds to Effort Expectancy from UTAUT. Relative Advantage from DOI relates to Performance Expectancy from UTAUT. From DOI to our study we added two new constructs: • Compatibility: measures the extent to which an innovation is perceived as aligned with the current consumer lifestyle, values, and past experiences. • Results Demonstrability is the degree to which the tangible results of using an innovation can be visible and communicated.	Tavares and Oliveira, 2018; Barney et al., 2020
CFIP	Published studies showed that privacy concerns with video consultation as a main topic for the patients. For this specific reason the Concern For Information Privacy (CFIP) framework is included in the study model.	Barney et al., 2020; Angst and Agarwal, 2009; Powell et al., 2017
Covid-19 Construct	Because the study took place during the Covid-19 pandemic period and following the same approach as in the literature a construct to measure the impact of Covid-19 was added in the model.	An et al., 2021

### 1.2 Research model

**Fig. 1:**
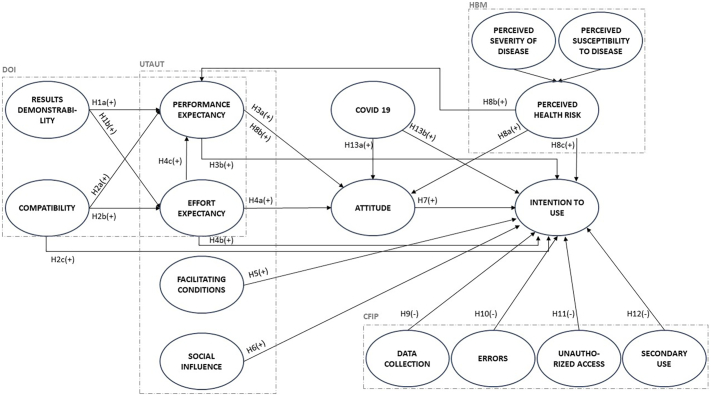
The research model.

Video consultations have been used for several years, albeit infrequently and without maximising their potential for patients and healthcare providers (Drake et al., 2021; Dekker et al., 2020). According to the most recent findings, the Covid-19 pandemic is changing this scenario, and people are more willing to use them (Drake et al., 2021; Dekker et al., 2020). By using mobile apps and internet platforms, the interaction between patients and their healthcare providers is transforming (Drake et al., 2021; Dekker et al., 2020). Studying video consultations' adoption encompasses understanding the different dimensions of the technology: an information communication technology with a novel use-case, potentially dealing with sensitive and confidential topics (Tavares and Oliveira, 2018; Angst and Agarwal, 2009; Drake et al., 2021; Dekker et al., 2020; Rogers, 1962). Therefore, to study video consultations, we need to incorporate information from different theories, covering all the previously mentioned dimensions (Fig. 1).

The diffusion of innovation theory (DOI) was identified, given the novelty of the technology. The unified theory of acceptance and use of technology (UTAUT) was chosen as it is commonly used in the information systems area. The health belief model was also chosen, as it captures the specificities of the healthcare sector well and has already shown promising results in this area (Kim and Park, 2012; Tavares and Oliveira, 2018; Zhang et al., 2015). Given the privacy concerns that arise in telehealth (Barney et al., 2020; Powell et al., 2017), the concerns for information privacy framework was used. A specific construct was incorporated to reflect the importance that COVID-19 had in society (Drake et al., 2021; Dekker et al., 2020).

Besides these main components of the model, two general demographic control variables were used: Age and Gender. One health-specific control variable was used, chronic disease, as literature supports that it has a significant impact (Kontos et al., 2014).

#### 1.2.1 Diffusion of innovation theory constructs

According to DOI, an innovation is an idea, process, or technology perceived as new or unfamiliar to individuals within a particular area or social system (Rogers, 1962). Five attributes of an innovation influence its adoption and diffusion - relative advantage, compatibility, complexity, trialability and observability (Rogers, 1962). The construct observability was divided into results demonstrability and visibility (Moore and Benbasat, 1991). Results demonstrability, compatibility, relative advantage, and complexity are going to be used. These last two, relative advantage and complexity, have equivalents in UTAUT, performance expectancy and effort expectancy. Relative advantage measures the degree to which consumers perceive benefits from using the technology (Rogers, 1962). The respective construct in UTAUT, performance expectancy, is defined as the degree to which consumers' use of a technology will provide benefits in performing associated activities (Viswanath et al., 2012). Complexity measures the degree to which an innovation is difficult to understand or to be used (Rogers, 1962). The respective construct in UTAUT, effort expectancy, is defined as the degree of ease associated with consumers' use of technology. As can be seen, each pair of constructs plays a similar role in the respective models, and both can positively influence intention to use (Moore and Benbasat, 1991). Trialability is defined as the degree to which an innovation may be experimented (Rogers, 1962). There is no evidence that our target population has participated in a trial usage of video consultations, therefore the construct was not used. Visibility was also not used as video consultations are personal experiences.

Results demonstrability is the degree to which the tangible results of using an innovation can be visible and communicated (Moore and Benbasat, 1991). There is evidence in the field of telemedicine stating that results demonstrability will positively influence effort expectancy and performance expectancy (Tavares and Oliveira, 2018).

H1a Results demonstrability will positively influence performance expectancy.

H1b Results demonstrability will positively influence effort expectancy.

Compatibility measures the extent to which an innovation is perceived as aligned with the current consumer lifestyle, values, and past experiences (Rogers, 1962). There is evidence in the field of telemedicine that compatibility will positively influence performance expectancy, effort expectancy and intention to use (Tavares and Oliveira, 2018; Zhang et al., 2015).

H2a Compatibility will positively influence performance expectancy.

H2b Compatibility will positively influence effort expectancy.

H2c Compatibility will positively influence intention to use.

#### 1.2.2 Unified theory of acceptance and use of technology constructs

UTAUT is frequently used in health care (Harst et al., 2019). UTAUT includes two dimensions mentioned before, performance expectancy and effort expectancy, adding facilitating conditions, social influence, intention to use and use behaviour. As video consultations have a low adoption rate (Roche. Mais de metade dos portugueses entende que pandemia dificultou o acesso à saúde. Accessed July 4, 2021), the use construct was not used. The Technology Acceptance Model (TAM) can be thought of as a simplified version of UTAUT^29^. It states that performance expectancy and effort expectancy impact attitude towards using a certain technology, which in its turn impacts the intention to use. Therefore, it is safe to incorporate the attitude construct from the TAM (Kim and Park, 2012; Venkatesh et al., 2003).

It was found that the equivalent construct in the UTAUT from the TAM, performance expectancy positively impacts attitude (Kim and Park, 2012; Ahadzadeh et al., 2015; An et al., 2021). The original UTAUT theory dictates that performance expectancy positively impacts intention to use (Venkatesh et al., 2003), and there is evidence that this effect holds in video consultations (Rho et al., 2015; Alam et al., 2020; Tavares and Oliveira, 2018).

H3a Performance expectancy will positively influence attitude.

H3b Performance expectancy will positively influence intention to use.

Evidence states that effort expectancy, positively impacts attitude and perceived usefulness (Kim and Park, 2012; An et al., 2021). The original UTAUT theory dictates that effort expectancy positively impacts intention to use (Venkatesh et al., 2003), and there is evidence that this effect holds in video consultations (Rho et al., 2015; Alam et al., 2020; Tavares and Oliveira, 2018).

H4a Effort expectancy will positively influence attitude.

H4b effort expectancy will positively influence intention to use.

H4c Effort expectancy will positively influence performance expectancy.

Facilitating conditions are the degree to which an individual believes that the necessary resources are available to support the use of the system (Viswanath et al., 2012). The original theory dictates that facilitating conditions influence intention to use positively (Venkatesh et al., 2003), as consumers with more facilitating conditions are more likely to have a higher intention to use a technology. Despite not always having a significant effect in the telemedicine field, some literature still supports that it may hold in the area (Alam et al., 2020).

H5 Facilitating conditions will positively influence intention to use.

Social influence is the degree to which an individual perceives that others important to him or her believe he or she should use the new system (Viswanath et al., 2012). The original UTAUT theory dictates that social influence positively influences intention to use (Venkatesh et al., 2003), as users tend to be influenced by others sharing similar constraints. According to literature, this hypothesis holds in telemedicine (Rho et al., 2015; Alam et al., 2020).

H6 Social influence will positively influence intention to use.

Attitude towards is the degree of good or bad evaluative affection associated with the consequences of using a system (Davis, 1985). The original TAM theory states that attitude positively influences intention to use [35], and this effect has also been proven in telemedicine (Kim and Park, 2012; Ahadzadeh et al., 2015; An et al., 2021).

H7 Attitude will positively influence intention to use.

#### 1.2.3 Health belief model

The HBM has shown promising results in telemedicine when used as a complement to other information system theories (Kim and Park, 2012; Ahadzadeh et al., 2015; Tavares and Oliveira, 2018; Yi et al., 2006). The HBM states that people are more motivated to act in healthy ways given the belief that they are susceptible to adverse health consequences (Carpenter, 2010). Literature shows that health beliefs and concerns indirectly affect the intention to use health information technology (Kim and Park, 2012). The construct perceived health risk comes from the HBM and was chosen as it suggests that belief in health risks predict the likelihood of engaging in health behaviour (Ahadzadeh et al., 2015). It is a second-order construct with perceived disease severity and susceptibility to a disease as their first order constructs. The perceived severity of a disease dimension refers to feelings about the clinical and social consequences of contracting an illness or leaving it untreated (Ahadzadeh et al., 2015). The perceived susceptibility to disease dimension refers to beliefs about the likelihood of getting a disease or condition (Ahadzadeh et al., 2015). Previous literature found that perceived health risk will have a direct positive impact on attitude towards and behavioral intention. The first findings are intuitive as the higher the perception of health risk, the more likely it is to have a positive attitude towards video consultations and to have a higher intention to use them. The literature also found that performance expectancy mediates the relationship between perceived health risk and attitude (Ahadzadeh et al., 2015).

H8a Perceived health risk will positively influence attitude.

H8b Performance expectancy mediates the relationship between perceived health risk and attitude.

H8c Perceived health risk will positively influence intention to use.

#### 1.2.4 Concerns for information privacy

Collection, errors, unauthorised access, and secondary use are the four dimensions from which the Concern For Information Privacy (CFIP) framework is constituted (Smith et al., 1996). This framework has been used in the context of telemedicine (Angst and Agarwal, 2009), and previous literature found that privacy is an area of concern for patients regarding video consultations (Barney et al., 2020; Powell et al., 2017).

Collection refers to the users' idea that companies are collecting an excessive amount of data (Angst and Agarwal, 2009). It is expected that collection will negatively influence intention to use.

H9 Collection will negatively impact intention to use.

Errors refer to the users' concerns of having incorrect data stored in databases (Angst and Agarwal, 2009). It is expected that errors will negatively influence intention to use.

H10 Errors will negatively impact intention to use.

Unauthorised Access refers to using users' personal information without permission (Angst and Agarwal, 2009). It is expected that unauthorised access will negatively influence intention to use.

H11 Unauthorised Access will negatively impact intention to use.

Secondary use of personal information refers to using users' personal information without their consent (Angst and Agarwal, 2009). It is expected that secondary use of personal information will negatively influence intention to use.

H12 Secondary Use of Personal Information will negatively impact intention to use.

#### 1.2.5 COVID-19 pandemic construct

Due to the change that the COVID-19 pandemic enforced in society, a COVID-19 related construct is used. Several constructs measuring anxiety and fear related to acceptance of technology have been tested in the context of COVID-19. However, there were no consistent or strong results in the studies (An et al., 2021; Al-Maroof et al., 2020; Kubb and Foran, 2020). Therefore, we included a single-item scale that evaluates if COVID-19, in a broader sense, was a driver of video consultations' acceptance.

H13a The COVID-19 pandemic will positively influence attitude.

H13b The COVID-19 pandemic will positively influence intention to use.

## 2 Methods

### 2.1 Measurement

The items were adapted from Venkatesh et al. (Viswanath et al., 2012; Venkatesh et al., 2003), Smith et al. and Angst et al. (Angst and Agarwal, 2009; Smith et al., 1996), Tavares and Oliveira (Tavares and Oliveira, 2018), and Ahadzadeh et al. (Ahadzadeh et al., 2015), with minor modifications to adapt to video consultations. The items are described in the Multimedia Appendix 1. The scale items were measured using a seven-point range scale from “1 – Strongly disagree” to “7- Strongly agree”. Social and demographic questions to characterise the sample were also made. Age was measured in years. Dummy variables were used for gender, 0 for female and 1 for male, and chronic diseases, 0 for not having any chronic disease and 1 for having one or more chronic diseases. The questionnaire was written in Portuguese. The items were translated from English to Portuguese, and a back-translation from Portuguese to English was made by a different translator and then compared to the original one to ensure the translation's correctness (Wild et al., 2005).

### 2.2 Data collection

Previous literature showed that younger people, who are more educated and have a higher health literacy, are more likely to use eHealth tools (Kontos et al., 2014; Or and Karsh, 2009). Most recent literature concerning video consultations identified that younger people with higher education are more knowledgeable and likely to be interested in using video consultations, pointing towards a potential digital divide (Drake et al., 2021; Dekker et al., 2020; Shaw et al., 2020). Due to the model's complexity, a minimum of 150 respondents was needed (Hair et al., 2017). Moreover, at least 184 respondents were needed to attain a statistical power of 80 % to detect R^2^ values of at least 0.10 with a 5 % probability of error (Hair et al., 2017; Cohen, 1992). Due to the need to have a meaningful sample size and respondents knowledgeable about the matter, the survey was distributed in two institutes of higher education in Lisbon. First, a pilot survey was conducted. It collected 11 responses, no issues were reported, and these responses were not included in the final analysis. Before the participants could answer the survey, an introduction about the research purpose was provided. Confidentiality and anonymity assurance was given to the participants. The participants had to state their consent to participate in the survey explicitly. An additional assurance was given to the participants stating if they decided not to complete the survey, the data collected would not be used and would be discarded. In total, 346 valid answers were obtained. It was possible to answer the survey through mobile phones and computers. The survey was available between the 27 April 2021 and 2 May 2021.

### 2.3 Data analysis

We decided to use partial least squares structural equation modeling (PLS-SEM) to test our model due to the model's complexity, the existence of many constructs, the existence of formatively measured constructs as part of the structural model, and our goal to identify the key driver constructs. These circumstances favour the use of PLS-SEM instead of covariance-based structural equation modeling (CB-SEM) (Hair et al., 2017). SmartPLS 3 statistical software was used to estimate the model (Hair et al., 2017). Before evaluating the structural model, we assessed the quality of the measurement model.

## 3 Results

### 3.1 Sample characteristics

The sample average age was approximately 35 years, and the majority of the respondents were women (60.7 %). The study sample is predominantly female (60 %). The academic institution from which we collected 81 % of the answers, 60 % of the students are female. That may explain the higher proportion of female responses (NovaSBE, 2020), (Table 2).

**Table 2:** Sample characteristics.

	Sample (number)	Sample (percentage)
Age (mean = 34.8)		
[18–34]	224	64.74
[35–49]	22	6.36
[50–64]	77	22.25
>64	23	6.65
Gender		
Male	136	39.31
Female	210	60.69
Education		
High School completed	23	6.64
Bachelor's Degree (ongoing or completed)	176	50.88
Master's Degree (ongoing or completed)	145	41.90
PhD (ongoing or completed)	2	0.58
Chronic disease		
Has one or more chronic disease	61	17.63
Does not have any chronic disease	285	82.37

### 3.2 Measurement model

As there are reflective and formative constructs, different measures to assess the quality of the model must be used. We start by evaluating the reflective constructs. The internal consistency measures how closely related the construct items are (Hair et al., 2017). To do so, we used Cronbach’s alpha and composite reliability. As seen in Table 3, all constructs display Cronbach’s alpha and composite reliability scores above 0.7, proof of the internal consistency (Hair et al., 2017). Item UA3, with an outer loading below 0.7, had to be dropped to achieve these values as it was worsening the model´s performance, leading to both Cronbach’s alpha and composite reliability values below the threshold (Hair et al., 2017). The convergent validity measures the degree to which one measure correlates positively with alternative measures of the same construct (Hair et al., 2017). It was assessed with the Average Variance Extracted (AVE) and outer loadings (Hair et al., 2017). AVE should be above 0.5. In Table 3, we can see that all constructs have a value above this threshold. The outer loadings should be above 0.7 (Hair et al., 2017). This criterion was also fulfilled, as depicted in Table 4. Discriminant validity is the length to which one construct is truthfully different from the others (Hair et al., 2017). It is assessed based on the cross-loadings and the Fornell and Larcker criterion. Specifically, an indicator's outer loading on the associated construct should be greater than any of its cross-loadings (Hair et al., 2017), as confirmed in the Multimedia Appendix 2. The Fornell and Larcker criterion affirms that the square root of AVE in each construct should be higher than any other correlation value among other constructs (Hair et al., 2017). This criterion holds for all constructs besides the second-order construct, PHR, as illustrated in Table 5. The correlation between PHR and PHR is lower than the correlation between PHR and PSE, and PHR and PSU, the first-order constructs that make up the higher-order one. This outcome happens because PHR is composed by PSE and PSU. Finally, the Heterotrait-monotrait ratio was analysed. It represents what the correlation between two constructs would be if they were measured perfectly (Hair et al., 2017). The confidence interval cannot include the value 1 (Hair et al., 2017), as confirmed in the Multimedia Appendix 2.

Perceived health risk is arranged as a reflective formative–type higher-order construct (Hair et al., 2017). We evaluated its multicollinearity according to the variance inflation factor (VIF), which denoted no collinearity issues as VIF values are below 5. The weights are positive and statistically significant, confirming the suitability of using this second-order construct (Hair et al., 2017).

**Table 3:** Cronbach’s alpha, composite reliability, and average variance extracted.

Constructs	Cronbach’s alpha	Composite reliability	Average variance extracted
Attitude	0.96	0.97	0.89
Compatibility	0.84	0.89	0.68
Data collection	0.94	0.96	0.89
Effort expectancy	0.91	0.94	0.8
Errors	0.91	0.93	0.76
Facilitating conditions	0.85	0.90	0.70
Intention to use	0.94	0.96	0.88
Perceived severity	0.85	0.90	0.70
Perceived susceptibility	0.86	0.90	0.61
Performance expectancy	0.90	0.93	0.77
Results demonstrability	0.80	0.88	0.71
Secondary use	0.85	0.85	0.58
Social influence	0.73	0.85	0.67
Unauthorised access	0.81	0.89	0.80

**Table 4:** Fornell and Larcker criterion.

Variables	AT	CO	CP	DC	EE	ER	FC	IU	PHR	PSE	PSU	PE	RD	SU	SI	UA	Age	CD	GD
AT	**0.94**																		
CO	−0.13	**1**																	
CP	0.80	0.56	**0.82**																
DC	−0.01	0.11	0.00	**0.94**															
EE	0.54	−0.21	0.49	−0.07	**0.89**														
ER	0.16	0.20	0.17	0.19	0.14	**0.87**													
FC	0.39	−0.22	0.30	−0.13	0.70	0.01	**0.83**												
IU	0.85	−0.08	0.75	0.04	0.49	0.14	0.38	**0.94**											
PHR	0.20	0.02	0.12	0.13	0.08	0.20	0.18	0.20	**0.68**										
PSE	0.22	0.02	0.15	0.07	0.16	0.22	0.19	0.21	0.77	**0.84**									
PSU	0.13	0.02	0.07	0.13	0.00	0.13	0.12	0.14	0.89	0.41	**0.78**								
PE	0.82	−0.12	0.81	0.04	0.49	0.14	0.34	0.80	0.20	0.22	0.13	**0.88**							
RD	0.58	0.04	0.57	0.08	0.49	0.11	0.34	0.57	0.14	0.15	0.09	0.60	**0.84**						
SU	0.10	0.10	0.05	0.09	0.15	0.31	0.15	0.13	0.13	0.19	0.05	0.08	0.10	**0.76**					
SI	0.55	−0.09	0.54	0.06	0.34	0.12	0.30	0.55	0.22	0.18	0.19	0.61	0.49	0.05	**0.82**				
UA	0.04	0.21	0.04	0.17	0.10	0.57	0.01	0.08	0.13	0.20	0.05	0.03	0.11	0.44	0.02	**0.9**			
Age	−0.13	−0.24	−0.12	0.11	−0.21	0.20	−0.22	−0.08	0.02	0.02	0.02	−0.12	0.04	0.11	−0.09	0.21	**1**		
CD	−0.03	0.36	−0.04	0.05	−0.02	0.13	−0.02	0.00	0.10	0.17	0.17	−0.03	−0.03	0.05	0.01	0.14	0.36	**1**	
GD	0.08	−0.11	0.09	0.06	0.16	−0.11	0.11	0.01	−0.07	−0.00	−0.00	0.02	0.05	−0.03	0.07	−0.14	−0.11	−0.09	**1**

AT = Attitude, CO = Covid-19, CP = Compatibility, DC = Data Collection, ER = Errors, EE = effort expectancy, FC = facilitating conditions, IU = intention to use, PE = performance expectancy, PSE = Perceiver Severity, PHR = Perceived Health Risk, PSU = Perceived Susceptibility, RD = Results Demonstrability, SI = social influence, SU = Secondary Use, UA = Unauthorised Access, CD = Chronic Disease, GD = Gender.

The diagonal is the square root of the reflective constructs' AVE, and the other values are the correlations between the constructs.

**Table 5:** Measurement model evaluation for the higher-order formative constructs.

Constructs	VIF	Weight	p-Value
Perceived Health Risk – Perceived Severity	1.20	0.50	<0.001
Perceived Health Risk – Perceived Susceptibility	1.20	0.69	<0.001

### 3.3 Structural model

The significance levels of the structural model path were estimated with a 5000 iterations bootstrap resampling to achieve maximum consistency in the results (Hair et al., 2017). As stated previously, the multicollinearity of all constructs was tested based on the VIF, and all values were below 5, which indicates no multicollinearity issues among the constructs. The R^2^ values were analysed to assess the structural model. The model explains 77.6 % of the variance in intention to use, 71.4 % in attitude, 70.1 % in performance expectancy, and 30.2 % in effort expectancy. Table 6 summarises the results of the structural model.

**Table 6:** Structural model results.

Dependent/independent variables	Beta	p-value	R^2^	R^2^ adjusted
IU			0.78	0.77
CP	0.089	0.152		
AT	**0.504**⁎⁎	**<0.001**		
PE	**0.196**⁎	**0.002**		
EE	−0.002	0.961		
FC	0.036	0.293		
SI	0.042	0.270		
CO	**0.151**⁎⁎	**<0.001**		
PHR	−0.003	0.908		
DC	0.043	0.174		
ER	−0.075	0.088		
UA	0.053	0.221		
SU	0.027	0.532		
Age	**0.064**⁎	**0.029**		
Gender	−0.036	0.192		
Chronic Disease	0.001	0.974		
AT			0.71	0.71
PE	**0.643**⁎⁎	**<0.001**		
EE	**0.138**⁎⁎	**0.001**		
CO	**0.170**⁎⁎	**<0.001**		
PHR	0.029	0.348		
Age	0.026	0.371		
Gender	0.046	0.128		
Chronic Disease	−0.013	0.677		
PE			0.70	0.70
PHR	**0.090**⁎	**0.002**		
RD	**0.159**⁎⁎	**<0.001**		
CP	**0.672**⁎⁎	**<0.001**		
EE	**0.081**⁎	**0.032**		
EE			0.30	0.30
RD	**0.312**⁎⁎	**<0.001**		
CP	**0.308**⁎⁎	**<0.001**		

AT = Attitude, CO = Covid-19, CP = Compatibility, DC = Data Collection, ER = Errors, EE = effort expectancy, FC = facilitating conditions, IU = intention to use, PE = performance expectancy, PSE = Perceiver Severity, PHR = Perceived Health Risk, PSU = Perceived Susceptibility, RD = Results Demonstrability, SI = social influence, SU = Secondary Use, UA = Unauthorised Access, CD = Chronic Disease, GD = Gender.

The diagonal is the square root of the reflective constructs' AVE, and the other values are the correlations between the constructs.

⁎⁎ *p* < 0.01.

⁎ *p* < 0.05.

We also evaluated the common method variance, using one of the most standard methods to assess it, namely Harman's one-factor test (Podsakoff et al., 2003). It states that if the total variance for any single factor is <50 %, common method variance should not be an issue (Podsakoff et al., 2003). The first factor, as expected, was the one with the greatest variance (30.4 %), still considerably lower than 50 %, reinforcing that common method variance should not be an issue. The marker-variable technique was also used, where an unrelated theoretical construct is used (Lindell and Whitney, 2001). No significant correlation was found between the research model constructs and the marker variable. Therefore, it can be concluded that common method variance was not a problem, verified by two different and established criteria.

## 4 Discussion

The results of our study support the use of an integrated model to cover all the different aspects of video consultation related technology adoption. The models, theories and dimensions used were: UTAUT, DOI, HBM, CFIP, and COVID-19. The model explained 77.6 % (R^2^) of the variance on intention to use. A good result was also achieved in Attitude, with an R^2^ of 71.4 %. The DOI theory proved useful, as 4 out of 5 hypotheses exclusively connected with DOI were supported (H1a→H2c). The COVID-19 construct also showed a relevant impact, having a statistically significant effect on both attitude and intention to use. UTAUT and HBM constructs also proved useful, notwithstanding a lower magnitude than the DOI and COVID-19 constructs. Regarding the CFIP framework, none of the constructs was found to be statistically significant. It seems that, in general, confidentiality concerns are not an issue for the adoption of video consultations.

### 4.1 Theoretical implications

As demonstrated in Table 7, the results demonstrability has a statistically significant effect on both performance expectancy and effort expectancy, supporting H1a(Results demonstrability will positively influence performance expectancy) and H1b(Results demonstrability will positively influence effort expectancy). This outcome suggests that the visibility and communicability of the results of using an innovation increase the perceived benefits and ease of use associated with video consultations. Compatibility has a statistically significant effect on performance expectancy and effort expectancy, supporting H2a(Compatibility will positively influence performance expectancy) and H2b(Compatibility will positively influence effort expectancy). However, no statistically significant effect in intention to use was found, not supporting H2c(Compatibility will positively influence intention to use). These findings evidence that when video consultations are perceived as aligned with the patient’s lifestyle, values, and past experiences, there is a higher perceived benefit and ease of use of the mentioned technology. It is interesting to note that this perceived alignment does not directly influence the intention to use. Literature supporting H1(a), H1(b), H2(a) and H2(b) exists (Tavares and Oliveira, 2018; Zhang et al., 2015).

**Table 7:** Hypothesis results.

Hypothesis	Path	Beta	p-Value	Result
**H1(a)**	RD to PE	0.159	<0.01	Supported
**H1(b)**	RD to EE	0.312	<0.01	Supported
**H2(a)**	CP to PE	0.672	<0.01	Supported
**H2(b)**	CP to EE	0.308	<0.01	Supported
H2(c)	CP to IU	0.089	0.152	Not Supported
**H3(a)**	PE to AT	0.643	<0.01	Supported
**H3(b)**	PE to IU	0.196	0.002	Supported
**H4(a)**	EE to AT	0.138	<0.01	Supported
H4(b)	EE to IU	−0.002	0.961	Not Supported
**H4(c)**	EE to PE	0.081	0.030	Supported
H5	FC to IU	0.036	0.293	Not Supported
H6	SI to IU	0.042	0.270	Not Supported
**H7**	AT to IU	0.504	<0.01	Supported
H8(a)	PHR to AT	0.029	0.348	Not Supported
**H8(b)**	(PHR to PE) * (PE to AT)	0.058	0.002	Supported
H8(c)	PHR to IU	−0.003	0.908	Not Supported
H9	DC to IU	0.043	0.174	Not Supported
H10	ER to IU	−0.075	0.088	Not Supported
H11	UA to IU	0.053	0.221	Not Supported
H12	SU to IU	0.027	0.532	Not Supported
**H13(a)**	CO to AT	0.170	<0.01	Supported
**H13(b)**	CO to IU	0.151	<0.01	Supported

AT = Attitude, CO = Covid-19, CP = Compatibility, DC = Data Collection, ER = Errors, EE = effort expectancy, FC = facilitating conditions, IU = intention to use, PE = performance expectancy, PHR = Perceived Health Risk, RD = Results Demonstrability, SI = social influence, SU = Secondary Use, UA = Unauthorised Access.

Bold indicates the Hypothesis is supported.

Performance expectancy has a statistically significant effect on attitude and intention to use, supporting H3a(Performance expectancy will positively influence attitude) and H3b(Performance expectancy will positively influence intention to use), and confirming that perceived benefits of video consultations are an important predictor of both attitude and intention to use. Several studies in specific areas related to telemedicine support H3(a) (Kim and Park, 2012; Ahadzadeh et al., 2015; An et al., 2021) and H3(b) (Rho et al., 2015; Alam et al., 2020; Tavares and Oliveira, 2018). Attitude had a statistically significant effect on intention to use, supporting H7(Attitude will positively influence intention to use). It confirms that the affection associated with using video consultations is an important predictor of the intention to use them. There are several studies in specific areas in telemedicine supporting H7 (Kim and Park, 2012; Ahadzadeh et al., 2015; An et al., 2021). Effort expectancy statistically impacted attitude and performance expectancy, supporting H4a(Effort expectancy will positively influence attitude) and H4b(effort expectancy will positively influence intention to use). However, there was no direct impact on intention to use, not supporting H4c(Effort expectancy will positively influence performance expectancy). The literature supports both H4(a) (Kim and Park, 2012; Ahadzadeh et al., 2015; An et al., 2021) and H4(b) (Kim and Park, 2012; An et al., 2021). Regarding H4(c), contradicting evidence exists. Some studies have proven this effect (Alam et al., 2020), while others have not proven it (Tavares and Oliveira, 2018; Yi et al., 2006; Dou et al., 2017). Facilitating conditions was not found statistically significant, not supporting H5(Facilitating conditions will positively influence intention to use). A possible explanation may be that patients already have all the resources and knowledge needed, not considering them a problem when deciding to use video consultations. Previous literature has already found this effect (Rho et al., 2015; Tavares and Oliveira, 2018), and it may be more prevalent during and after the COVID-19 pandemic, where many people increased their use of video conferencing platforms. Social influence was not found statistically significant, not supporting H6(Social influence will positively influence intention to use). Contradictory studies exist regarding this impact (Rho et al., 2015; Alam et al., 2020; Tavares and Oliveira, 2018). The private nature of consultations may explain why patients are not influenced by the opinion of others.

The suggested mediation from perceived health risk to attitude through performance expectancy was found to have a full effect as only the indirect impact was found statistically significant, meaning that H8b(Performance expectancy mediates the relationship between perceived health risk and attitude) was supported, and H8a(Perceived health risk will positively influence attitude) and H8c(Perceived health risk will positively influence intention to use) were not supported. Previous literature supports this effect (Kim and Park, 2012; Ahadzadeh et al., 2015).

None of the CFIP constructs was found statistically significant, not supporting H9(Collection will negatively impact intention to use), H10(Errors will negatively impact intention to use), H11(Unauthorised Access will negatively impact intention to use), and H12(Secondary Use of Personal Information will negatively impact intention to use). A possible explanation is that a wide array of topics can be discussed with the physician, ranging from sensitive to familiar topics. If the consultation focuses on psychology or infectious diseases, the effect may be found statistically significant. If the consultation focuses on general health topics, the non-significant effect we reached may be expected, as users may not worry about confidentiality.

Previous literature could not prove the relationship between COVID-19 and video consultations (An et al., 2021). Our study shows that the COVID-19 pandemic influenced both the attitude and the intention to use video consultations, supporting H13a(The COVID-19 pandemic will positively influence attitude) and H13b(The COVID-19 pandemic will positively influence intention to use). These results are reinforced by recent statistics that show the increase in the ubiquity of video consultations' during the pandemic (Mann et al., 2020; Dubin et al., 2020; Rizzi et al., 2020). This study shows a potential effect of the COVID-19 pandemic in the attitude and intention to use this technology.

Regarding the control variables, only age was found to have a statistically significant effect on intention to use. Contrarily to popular belief, age was found to impact intention to use positively, meaning that older people were more likely to have a higher intention to use video consultations. These results are reinforced by previous literature that suggests higher age implies a more frequent need for health services (Tavares and Oliveira, 2016).

### 4.2 Managerial implications

Two different organizational areas can significantly benefit from this study, namely those related to developing video consultation platforms and those responsible for creating marketing communication strategies. Before addressing the specific findings, the most important general one is that COVID-19 positively influenced the intention to use video consultations. The effect was widely talked about in the public sphere, and this study brings evidence to support it.

With the health services overwhelmed with serious patients, most of them older, less severe patients were encouraged during Covid pandemic to use more eHealth tools to communicate with healthcare providers (Morais et al., 2022; Starke et al., 2021). In general, younger people have less severe Covid and also have less severe diseases (Starke et al., 2021). Even if during the peak of the pandemic the group with higher usage of video consultations were the younger ones, those can help reducing the access of people with less severe diseases to the overwhelmed services during Covid (Morais et al., 2022; Starke et al., 2021).

According to DOI an IT adoption is the initial use of a new technology, whereas IT usage is the subsequent continued use of a new or innovative technology, and may be influenced by different drivers (Zhang et al., 2015; Karahanna and Straub, 1999). According to DOI theory, Covid-19 acted as strong driver of adoption, but to promote continuous usage and implement new video consultation projects, the other drivers in the model should be the ones considered in future implementations. Apart of the Covid-19 construct, all other constructs from DOI showed to be statistically relevant, in line with DOI theory (Zhang et al., 2015; Karahanna and Straub, 1999). This study identified relevant managerial insights that can be used beyond the pandemic phase.

Implications at the platform development level can be derived from the compatibility construct when we identify the importance of compatibility with past experiences. Hospitals and other healthcare entities should develop platforms with a similar interface, functionalities, and experience to the most widely used video conferencing platforms. By doing so, perceived productivity and ease of use will increase, positively influencing users' attitudes and intention to use video consultations. An interface that emphasises time savings and any other result that derives from using video consultations should also be accessible to users. It will increase perceived productivity derived from video consultations while decreasing perceived effort, therefore positively influencing attitude and intention to use them. Despite the non-importance of the CFIP constructs, all the privacy regulations should be fulfilled when developing platforms. In short, the higher the productivity gains and the easier it is to use these platforms, the better the attitude towards video consultations is and the higher the intention to use them. Developers must find possible ways to target these two areas directly while also looking to increase the similarity with previously used video conferencing tools and displaying the results derived from the consultations. These two factors will positively influence both perceived productivity gains and easiness of using video consultations.

Implications for communication strategies can be derived from most constructs. They will be analysed in this paragraph. Regarding compatibility, providers should find how video consultations can align with the users' lifestyles, values, and past experiences, emphasising these when developing communication strategies. For example, a specific segment of patients between 18 and 30 years old and digital natives can be identified, and a highlight of how video consultations fit their lives should be made. Addressing the importance of results demonstrability, providers must clearly express the results of using video consultations. By doing so, patients will understand the results obtained from video consultations more clearly, leading to higher expected productivity and lower perceived effort. Video consultations lead to time savings that can reach 3 h per visit. This factor exemplifies one result that can be communicated to users (Powell et al., 2018). These time savings arise from different aspects, such as no more need for waiting times and time-consuming commutes to hospitals (Powell et al., 2018). There is a direct impact from effort expectancy on attitude. Therefore, it is essential to show that most people already have the resources and knowledge needed to use video consultations. Based on performance expectancy, the communication strategy must also highlight the benefits of using video consultations, particularly the increase in productivity they can expect. Some routine visits that lead to a high time expenditure do not require the patient to be physically present, leading to no need to go to the hospital and easier access to experts. From the perceived health risk construct, we first realise the positive influence on attitude mediated by performance expectancy, and secondly on intention to use mediated by performance expectancy. This aspect means that people who are more concerned about their health have higher perceived productivity gains from using a platform that provides them possible ways to solve their concerns. Patients more concerned with their health will perceive the platform as a more effective and a more productive means of communicating with their healthcare professional than patients less concerned about their health. This phenomenon makes it even more important to address the productivity gains that can be expected from using video consultations. As important as understanding the most important drivers of adoption, is evidencing what may have been thought of as a driver but is not. The CFIP constructs have shown that privacy concerns are not important for users to adopt video consultations, therefore minor emphasis should be put on the communication strategy.

### 4.3 Limitations and future research

The model complexity led to the need of having a large number of respondents to detect R^2^ values with a statistical power of 80 %. Due to the low adoption rate reported in the literature (Roche. Mais de metade dos portugueses entende que pandemia dificultou o acesso à saúde. Accessed July 4, 2021), a sample with people knowledgeable about video consultations had to be selected, which led to the distribution of the survey in two higher education institutions in Lisbon, as people with higher education tend to use more e-health and video consultations (Drake et al., 2021; Dekker et al., 2020). Nonetheless, recently published studies indicate that the use of video consultations has increased (Jiménez-Rodríguez et al., 2020; Dubin et al., 2020; Rizzi et al., 2020). Future studies should include a more representative general population sample and include the Use construct based on these findings.

With the proof that COVID-19 impacted the intention to use video consultations, future qualitative research can look deeper into this area to understand the root causes. Now that this study has evaluated video consultations as a whole, it is essential to try to understand the adoption and use in specific healthcare areas (general practice, oncology, psychology, etc.) because the drivers of use can be different between a patient with a mental health disorder, an infectious disease, or a simple follow-up appointment. Cross country comparisons and comparisons between different demographic groups from the same country to evaluate the differences in the drivers of adoption can also represent crucial succeeding research. Lastly, future research could also focus on understanding the drivers of using video consultations from the healthcare provider perspective. Not only are patients critical to the adoption of this technology, but physicians and other healthcare providers should also believe that video consultations can be a powerful tool to communicate with their patients.

This study sample didn’t focus particularly on older people. It is known that older adults have more difficulties with digital technologies including healthcare related technologies (Jacob et al., 2022; Pew Research Center, 2014). Specific studies targeting this population and video consultations adoption may provide relevant insights.

## 5 Conclusion

Video consultations are a promising part of telemedicine with the potential to help solve some of the imminently arising healthcare challenges, which will undoubtedly intensify in the future. Our respondents comprised primarily young adults who had a complete university degree or ongoing tertiary studies. The most significant constructs were performance expectancy, effort expectancy, compatibility, the COVID-19 pandemic, and perceived health risk. All the theories besides CFIP had significant constructs, which shows that DOI, UTAUT, and HBM are important to explain the adoption of video consultations. In addition to the significance of the constructs, the model achieved strong R^2^ results, explaining 77.6 % of the variance in intention to use and 71.4 % of the variance in attitude.

Firstly, this study shows that the COVID-19 pandemic impacted the intention to use video consultations indeed. It also brings evidence for the outline of platform development and communication strategies that can lead to a higher adoption by users. The need to make platforms similar to previous ones used by the target audience is evident. Moreover, communicating the suitability of this technology to the lifestyle and past experiences of the user is equally imperative. Emphasising the results of using video consultations is hugely relevant as well. Of the utmost importance is to increase the perceived productivity gains users can achieve through video consultations and their perceived ease of use, as these tangible benefits will positively impact the intention to use the technology and the attitudes of the users towards it. Furthermore, it will make patients more concerned about their health have even higher perceived productivity gains from using the technology.

With this study, we built upon the previous knowledge regarding patients' adoption of video consultations, evidencing the importance of the COVID-19 pandemic, perceived health risks, and compatibility. Important implications for healthcare entities aiming to implement video consultation tools were also extracted. The investigation also shows that high explanatory power can be achieved by using specific constructs from the different areas that compromise technology and use cases.

The following are the supplementary data related to this article.

Questionnaire items Questionnaire items

Supplementary statistics: outer loadings, cross loadings, and confidence intervals for HTMT Supplementary statistics: outer loadings, cross loadings, and confidence intervals for HTMT

## Funding

This work was supported by national funds through FCT (Fundação para a Ciência e a Tecnologia) under the project - UIDB/04152/2020 - Centro de Investigação em Gestão de Informação (MagIC).

## Declaration of competing interest

The authors declare that they have no known competing financial interests or personal relationships that could have appeared to influence the work reported in this paper.
