# Supplementary material for: Adoption of video consultations during the COVID-19 pandemic
Source: Internet Interv. 2023 Jan 20;31:100602. doi: 10.1016/j.invent.2023.100602 (PMC9852263; doi:10.1016/j.invent.2023.100602)
Supplement: Questionnaire items [file mmc1.docx]

**Multimedia Appendix A**

| **Constructs** | **Items** | **Sources** |
| --- | --- | --- |
| **Results Demonstrability** | RD1 - I would have no difficulty telling others about the results of using a Video Consultation | 1 |
|  | RD2 - I believe I could communicate to others the consequences of using a Video Consultation |  |
|  | RD3 - The results of using Video Consultations are apparent to me |  |
| **Compatibility** | CP1 - Using a Video Consultation is compatible with all aspects of my health care | 1 |
|  | CP2 - Using a Video Consultation is compatible with my current situation |  |
|  | CP3 - I think that using a Video Consultation fits well with the way I like to manage my health care |  |
|  | CP4 - Using Video Consultations fits in my lifestyle |  |
| **Performance Expectancy** | PE1 – I consider Video Consultations would be useful in my daily life | 2, 3 |
|  | PE2 – Using Video Consultations increases my chances of achieving things that are important to me |  |
|  | PE3 – Using Video Consultations helps me accomplish health-care related objectives more quickly |  |
|  | PE4 – Using Video Consultations increases my productivity |  |
| **Effort Expectancy** | EE1 – Learning how to use Video Consultations would be easy for me | 2, 3 |
|  | EE2 – My interaction with Video Consultations would be clear and understandable |  |
|  | EE3 – I find that it would be easy to use Video Consultations |  |
|  | EE4 – It would be easy for me to become skillful at using Video Consultations |  |
| **Facilitating Conditions** | FC1 – I have the resources necessary to use Video Consultations | 2, 3 |
|  | FC2 – I have the knowledge necessary to use Video Consultations |  |
|  | FC3 – Video Consultations is compatible with other technologies I use |  |
|  | FC4 – I can get help from others when I have difficulties using Video Consultations |  |
| **Social Influence** | SI1 – People who are important to me think that I should use Video Consultations | 2, 3 |
|  | SI2 – People who influence my behavior think that I should use Video Consultations |  |
|  | SI3 – People whose opinions I value prefer that I use Video Consultations |  |
| **Attitude Towards** | AT1 - Using Video Consultations for my health care would be a good idea | 4 |
|  | AT2 - Using Video Consultations for my health care would be a wise idea |  |
|  | AT3 - I like the idea of using Video Consultations for my health care |  |
|  | AT4 - Using Video Consultations for my health care would be a pleasant experience |  |
| **Intention to Use** | IU1 – I intent to use Video Consultations in the future | 2, 3 |
|  | IU2 – I will try to use Video Consultations whenever it is possible |  |
|  | IU3 – I plan to use Video Consultations frequently |  |
| **COVID-19** | CO1 – I consider that the COVID-19 pandemic increased my propensity to use Video Consultations |  |
| **Perceived Severity of Disease** | PSE1 – I am afraid of having a new serious disease or the deterioration of an existing one | 4, 1 |
|  | PSE2 – If I face a serious disease or a deterioration of an existing one, I will have difficulties with my work like or domestic affairs |  |
|  | PSE3 – If I face a serious disease or a deterioration of an existing one, it will hinder my personal relationships |  |
|  | PSE4 - If I face a serious disease or a deterioration of an existing one, I will be long haunted by resultant problems |  |
| **Perceived Susceptibility to Disease** | PSU1 – There is a high probability that I will be exposed to a serious disease | 4, 1 |
|  | PSU2 – There is a person with a serious disease among my family members |  |
|  | PSU3 – It is highly likely that I will have a serious disease in my lifetime |  |
|  | PSU4 – I have a higher likelihood of having a serious disease |  |
|  | PSU5 – I have a strong possibility of facing a serious disease or the deterioration of an existing one due to improper daily habits (drinking, smoking, dietary habits, lack of exercise, etc.) |  |
|  | PSU6 – I would say I am the type of person who is likely to get a serious disease |  |
| **Data Collection** | DC1 – It usually bothers me when health care entities ask for my personal information | 5, 6 |
|  | DC2 – When health care entities ask me for personal information, I sometimes think twice before providing it |  |
|  | DC3 – It bothers me to give personal information to so many companies |  |
| **Errors** | ER1 – All the personal information in computer databases should be double-checked for accuracy, no matter how much this costs | 5, 6 |
|  | ER2 – Health care entities should take more steps to make sure that the personal information in their files is accurate |  |
|  | ER3 – Health care entities should have better procedures to correct errors in personal information |  |
|  | ER4 – Health care entities should devote more time and effort to verifying the accuracy of the personal information in their databases |  |
| **Unauthorized Access** | UA1 – Health care entities should devote more time and effort to preventing unauthorized access to personal information | 5, 6 |
|  | UA2 – Computer databases that contain personal information should be protected from unauthorized access, no matter how much it costs |  |
|  | ^a^UA3 – Health care entities should never share personal information with other health care entities unless it has been authorized by the individuals who provided the information^*^ |  |
| **Secondary Use** | SU1 – Health care entities should not use personal information for any purpose unless it has been authorized by the individuals who provided the information | 5, 6 |
|  | SU2 – When people give personal information to a health care entity for some reason, the entity should never use the information for any other reason |  |
|  | SU3 – Health care entities should never sell the personal information in their computer databases to other health care entities |  |
|  | SU4 – Health care entities should never share personal information with other entities unless it has been authorized by the individuals who provided the information |  |

**^a^** Item UA3 was dropped from the analysis, with an outer loading below 0.7, as it was worsening the model´s performance, leading to both Cronbach’s alpha and composite reliability values below the threshold.

References

1. Tavares J, Oliveira T. New integrated model approach to understand the factors that drive electronic health record portal adoption: Cross-sectional national survey. J Med Internet Res. 2018;20(11):1-17. doi:10.2196/11032

2. Viswanath V, James Y, Thong L, Xin X. Consumer acceptance and use of information technology: Extending the unified theory of acceptance and use of technology. Encephale. 2012;53(1):59-65. https://www.jstor.org/stable/41410412

3. Venkatesh V, Morris MG, Davis GB, Davis FD. User acceptance of information technology: toward a unified view. 2003;27(3):425-478.

4. Ahadzadeh AS, Pahlevan Sharif S, Ong FS, Khong KW. Integrating health belief model and technology acceptance model: An investigation of health-related internet use. J Med Internet Res. 2015;17(2):1-17. doi:10.2196/jmir.3564

5. Angst CM, Agarwal R. Adoption of electronic health records in the presence of privacy concerns: The elaboration likelihood model and individual persuasion. Proc 15th Int Conf Inf Qual. 2009;27(3):218-228. http://dx.doi.org/10.1016/j.soncn.2011.04.007

6. Smith HJ, Milberg SJ, Burke SJ. Information privacy: Measuring individuals’ concerns about organizational practices. MIS Q Manag Inf Syst. 1996;20(2):167-195. doi:10.2307/249477
