# Supplementary material for: Adoption of video consultations during the COVID-19 pandemic
Source: Internet Interv. 2023 Jan 20;31:100602. doi: 10.1016/j.invent.2023.100602 (PMC9852263; doi:10.1016/j.invent.2023.100602)
Supplement: Supplementary statistics: outer loadings, cross loadings, and confidence intervals for HTMT [file mmc2.docx]

**Multimedia Appendix 2**

Table B.2.1. Outer Loadings and Cross Loadings, ^a^

| Constructs | AT | CP | DC | ER | EE | FC | IU | PE | PSE | PSU | RD | SI | SU | UA |
| --- | --- | --- | --- | --- | --- | --- | --- | --- | --- | --- | --- | --- | --- | --- |
| Items |  |  |  |  |  |  |  |  |  |  |  |  |  |  |
| AT1 | **.94** | .74 | .01 | .16 | .49 | .35 | .77 | .75 | .23 | .15 | .54 | .54 | .09 | .07 |
| AT2 | **.94** | .73 | -.03 | .15 | .48 | .36 | .75 | .75 | .23 | .11 | .52 | .53 | .10 | .02 |
| AT3 | **.94** | .78 | .01 | .13 | .51 | .37 | .85 | .81 | .21 | .13 | .55 | .51 | .08 | .01 |
| AT4 | **.95** | .76 | -.02 | .17 | .53 | .39 | .82 | .78 | .18 | .11 | .58 | .51 | .09 | .04 |
| CP1 | .47 | **.72** | .06 | .12 | .28 | .09 | .42 | .48 | .14 | -.06 | .36 | .39 | .01 | .03 |
| CP2 | .59 | **.79** | -.05 | .15 | .41 | .27 | .52 | .57 | .10 | -.05 | .48 | .38 | .06 | .10 |
| CP3 | .74 | **.88** | .03 | .16 | .41 | .28 | .72 | .76 | .10 | .13 | .51 | .50 | .05 | .00 |
| CP4 | .76 | **.89** | -.01 | .13 | .47 | .31 | .73 | .80 | .16 | .14 | .52 | .49 | .04 | .02 |
| DC1 | -.02 | -.01 | **.95** | .15 | -.09 | -.14 | .04 | .03 | .04 | .13 | .07 | .05 | .08 | .13 |
| DC2 | .02 | .02 | **.96** | .20 | -.04 | -.10 | .04 | .06 | .08 | .11 | .09 | .08 | .09 | .15 |
| DC3 | -.03 | -.01 | **.91** | .22 | -.07 | -.14 | .02 | .03 | .09 | .15 | .07 | .04 | .11 | .24 |
| ER1 | .17 | .20 | .24 | **.90** | .13 | -.02 | .16 | .14 | .20 | .10 | .12 | .14 | .31 | .47 |
| ER2 | .08 | .08 | .14 | **.83** | .11 | .04 | .04 | .07 | .19 | .10 | .06 | .07 | .28 | .51 |
| ER3 | .11 | .08 | .13 | **.86** | .13 | .05 | .06 | .08 | .18 | .13 | .06 | .03 | .29 | .54 |
| ER4 | .15 | .15 | .11 | **.90** | .13 | .02 | .12 | .13 | .21 | .13 | .11 | .11 | .22 | .54 |
| EE1 | .41 | .39 | -.07 | .08 | **.87** | .64 | .39 | .4 | .14 | .01 | .41 | .26 | .13 | .09 |
| EE2 | .57 | .53 | -.08 | .15 | **.91** | .57 | .51 | .52 | .13 | -.06 | .51 | .39 | .12 | .07 |
| EE3 | .45 | .39 | -.06 | .15 | **.91** | .64 | .40 | .39 | .14 | .02 | .40 | .26 | .10 | .08 |
| EE4 | .46 | .39 | -.05 | .13 | **.88** | .65 | .44 | .42 | .15 | .07 | .41 | .28 | .17 | .11 |
| FC1 | .33 | .25 | -.08 | .04 | .61 | **.86** | .29 | .26 | .18 | .06 | .27 | .22 | .17 | .01 |
| FC2 | .33 | .29 | -.10 | -.05 | .61 | **.88** | .35 | .30 | .14 | .17 | .30 | .23 | .08 | -.05 |
| FC3 | .3 | .27 | -.11 | -.02 | .61 | **.84** | .30 | .29 | .14 | .08 | .26 | .24 | .15 | .05 |
| FC4 | .34 | .20 | -.14 | .08 | .50 | **.75** | .30 | .26 | .17 | .08 | .30 | .30 | .13 | .04 |
| IU1 | .85 | .73 | .02 | .11 | .50 | .41 | **.94** | .79 | .18 | .13 | .58 | .54 | .09 | .06 |
| IU2 | .77 | .67 | .03 | .14 | .43 | .31 | **.95** | .71 | .21 | .13 | .52 | .46 | .14 | .10 |
| IU3 | .77 | .71 | .06 | .13 | .45 | .33 | **.94** | .75 | .20 | .14 | .52 | .54 | .14 | .08 |
| PE1 | .79 | .78 | -.01 | .09 | .52 | .35 | .79 | **.87** | .18 | .07 | .57 | .49 | .08 | .03 |
| PE2 | .70 | .72 | .08 | .15 | .38 | .26 | .69 | **.90** | .20 | .15 | .50 | .59 | .07 | .01 |
| PE3 | .74 | .73 | .04 | .16 | .42 | .28 | .68 | **.91** | .21 | .16 | .53 | .57 | .09 | .04 |
| PE4 | .63 | .60 | .04 | .07 | .39 | .28 | .62 | **.83** | .19 | .08 | .48 | .50 | .05 | .01 |
| PSE1 | .17 | .14 | .03 | .22 | .13 | .13 | .16 | .17 | **.71** | .42 | .15 | .18 | .21 | .16 |
| PSE2 | .16 | .10 | .07 | .17 | .09 | .14 | .15 | .17 | **.90** | .34 | .10 | .11 | .15 | .19 |
| PSE3 | .17 | .11 | .09 | .14 | .10 | .13 | .16 | .16 | **.83** | .26 | .09 | .11 | .12 | .14 |
| PSE4 | .25 | .16 | .05 | .21 | .20 | .23 | .22 | .24 | **.90** | .33 | .15 | .18 | .16 | .18 |
| PSU1 | .08 | .05 | .14 | .12 | .02 | .11 | .11 | .11 | .43 | **.74** | .13 | .21 | .09 | .08 |
| PSU2 | .14 | .11 | .11 | .10 | .08 | .14 | .14 | .15 | .19 | **.60** | .07 | .12 | -.03 | .04 |
| PSU3 | .07 | .01 | .07 | .06 | .00 | .12 | .08 | .08 | .32 | **.85** | .07 | .15 | .05 | .04 |
| PSU4 | .11 | .04 | .07 | .12 | .00 | .10 | .11 | .08 | .33 | **.91** | .05 | .17 | .05 | .05 |
| PSU5 | .15 | .10 | .13 | .11 | -.05 | .04 | .12 | .13 | .27 | **.68** | .04 | .11 | .04 | .01 |
| Constructs | AT | CP | DC | ER | EE | FC | IU | PE | PSE | PSU | RD | SI | SU | UA |
| Items |  |  |  |  |  |  |  |  |  |  |  |  |  |  |
| PSU6 | .10 | .03 | .11 | .10 | -.03 | .06 | .12 | .09 | .32 | **.85** | .07 | .12 | .03 | .02 |
| RD1 | .59 | .55 | .05 | .11 | .49 | .31 | .56 | .58 | .16 | .07 | **.89** | .43 | .11 | .09 |
| RD2 | .33 | .35 | .05 | .14 | .36 | .29 | .32 | .39 | .12 | .04 | **.81** | .37 | .12 | .18 |
| RD3 | .52 | .53 | .11 | .05 | .37 | .26 | .53 | .52 | .09 | .12 | **.83** | .43 | .02 | .03 |
| SI1 | .51 | .50 | .06 | .13 | .31 | .24 | .51 | .56 | .18 | .17 | .48 | **.93** | .07 | .05 |
| SI2 | .51 | .49 | .06 | .11 | .32 | .30 | .50 | .58 | .17 | .19 | .46 | **.96** | .03 | .02 |
| SI3 | .54 | .53 | .08 | .13 | .31 | .27 | .54 | .58 | .17 | .19 | .46 | **.95** | .05 | .03 |
| SU1 | .06 | .03 | .05 | .32 | .14 | .18 | .06 | .05 | .21 | .00 | .07 | -.02 | **.76** | .44 |
| SU2 | .02 | .00 | .01 | .27 | .11 | .10 | .04 | .02 | .20 | .04 | .06 | -.06 | **.71** | .38 |
| SU3 | .01 | -.03 | -.02 | .25 | .13 | .12 | -.02 | -.01 | .16 | .01 | .03 | -.03 | **.60** | .30 |
| SU4 | .10 | .05 | .11 | .25 | .13 | .12 | .14 | .09 | .14 | .06 | .09 | .09 | **.94** | .36 |
| UA1 | .04 | .05 | .18 | .55 | .09 | .01 | .09 | .03 | .18 | .06 | .12 | .04 | .40 | **.98** |
| UA2 | .00 | .00 | .09 | .50 | .11 | .01 | .03 | .00 | .21 | .03 | .07 | -.05 | .48 | **.80** |

^a^AT = Attitude, CP = Compatibility, DC = Data Collection, ER = Errors, EE = Effort Expectancy, FC = Facilitating Conditions, IU = Intention to Use, PE = Performance Expectancy, PSE = Perceiver Severity, PSU = Perceived Susceptibility, RD = Results Demonstrability, SI = Social Influence, SU = Secondary Use, UA = Unauthorised Access

**Table B.2.2.** Confidence Intervals for HTMT. Average HTMT values computed from 5000 bootstrap samples.

| ^b^ | Original Sample (O) | Sample Mean (M) | 2,5%^a^ | 97,5%^a^ |
| --- | --- | --- | --- | --- |
| CO -> AT | .64 | .64 | .56 | .71 |
| CP -> AT | .87 | .87 | .83 | .91 |
| CP -> CO | .59 | .59 | .49 | .68 |
| DC -> AT | .02 | .06 | .02 | .14 |
| DC -> CO | .05 | .07 | .01 | .17 |
| DC -> CP | .05 | .08 | .04 | .15 |
| EE -> AT | .57 | .56 | .48 | .64 |
| EE -> CO | .47 | .47 | .36 | .58 |
| EE -> CP | .54 | .54 | .43 | .63 |
| EE -> DC | .08 | .09 | .03 | .20 |
| ER -> AT | .16 | .16 | .06 | .27 |
| ER -> CO | .12 | .13 | .04 | .24 |
| ER -> CP | .17 | .17 | .08 | .28 |
| ER -> DC | .20 | .02 | .09 | .31 |
| ER -> EE | .15 | .16 | .06 | .28 |
| FC -> AT | .43 | .43 | .32 | .53 |
| FC -> CO | .44 | .44 | .32 | .55 |
| FC -> CP | .34 | .34 | .22 | .45 |
| FC -> DC | .15 | .15 | .05 | .27 |
| FC -> EE | .79 | .79 | .69 | .88 |
| FC -> ER | .08 | .10 | .06 | .17 |
| IU -> AT | .89 | .89 | .85 | .92 |
| IU -> CO | .67 | .67 | .59 | .74 |
| IU -> CP | .82 | .82 | .76 | .87 |
| IU -> DC | .04 | .07 | .02 | .16 |
| IU -> EE | .53 | .52 | .44 | .61 |
| IU -> ER | .12 | .13 | .05 | .24 |
| IU -> FC | .42 | .42 | .32 | .51 |
| PSE -> AT | .25 | .25 | .13 | .37 |
| PSE -> CO | .26 | .26 | .13 | .38 |
| PSE -> CP | .18 | .19 | .08 | .31 |
| PSE -> DC | .08 | .10 | .03 | .21 |
| PSE -> EE | .18 | .18 | .08 | .31 |
| PSE -> ER | .25 | .25 | .12 | .38 |
| PSE -> FC | .22 | .22 | .10 | .35 |
| PSE -> IU | .23 | .23 | .11 | .36 |
| PSU -> AT | .15 | .16 | .07 | .26 |
| PSU -> CO | .17 | .17 | .07 | .27 |
| PSU -> CP | .14 | .16 | .11 | .23 |
| PSU -> DC | .16 | .16 | .07 | .28 |
| PSU -> EE | .07 | .10 | .06 | .17 |
| PSU -> ER | .15 | .16 | .07 | .27 |
| PSU -> FC | .14 | .16 | .08 | .27 |
| PSU -> IU | .17 | .17 | .07 | .28 |
| PSU -> PSE | .47 | .47 | .36 | .56 |
| PE -> AT | .88 | .88 | .84 | .91 |
| PE -> CO | .65 | .65 | .57 | .72 |
| PE -> CP | .90 | .90 | .86 | .94 |
| PE -> DC | .06 | .08 | .03 | .16 |
| PE -> EE | .53 | .53 | .44 | .62 |
| PE -> ER | .13 | .14 | .06 | .26 |
| PE -> FC | .38 | .38 | .27 | .49 |
| PE -> IU | .86 | .86 | .82 | .90 |
| PE -> PSE | .25 | .26 | .13 | .38 |
| PE -> PSU | .16 | .17 | .09 | .28 |
| RD -> AT | .65 | .65 | .56 | .72 |
| RD -> CO | .45 | .45 | .34 | .55 |
| RD -> CP | .68 | .68 | .59 | .76 |
| RD -> DC | .09 | .10 | .04 | .20 |
| RD -> EE | .56 | .56 | .45 | .66 |
| RD -> ER | .12 | .14 | .06 | .25 |
| RD -> FC | .41 | .41 | .28 | .53 |
| RD -> IU | .65 | .65 | .56 | .73 |
| RD -> PSE | .18 | .18 | .08 | .32 |
| RD -> PSU | .11 | .14 | .07 | .24 |
| RD -> PE | .69 | .69 | .61 | .76 |
| SU -> AT | .06 | .08 | .03 | .17 |
| SU -> CO | .06 | .09 | .03 | .17 |
| SU -> CP | .06 | .09 | .05 | .15 |
| SU -> DC | .07 | .09 | .04 | .16 |
| SU -> EE | .18 | .18 | .07 | .31 |
| SU -> ER | .39 | .39 | .27 | .50 |
| SU -> FC | .19 | .19 | .08 | .33 |
| SU -> IU | .09 | .11 | .05 | .18 |
| SU -> PSE | .25 | .25 | .14 | .37 |
| SU -> PSU | .07 | .10 | .06 | .16 |
| SU -> PE | .07 | .09 | .05 | .16 |
| SU -> RD | .10 | .12 | .06 | .22 |
| SI -> AT | .65 | .64 | .54 | .73 |
| SI -> CO | .51 | .50 | .38 | .61 |
| SI -> CP | .66 | .66 | .56 | .76 |
| SI -> DC | .11 | .13 | .05 | .25 |
| SI -> EE | .44 | .44 | .31 | .56 |
| SI -> ER | .20 | .21 | .11 | .33 |
| SI -> FC | .42 | .42 | .30 | .54 |
| SI -> IU | .63 | .63 | .52 | .73 |
| SI -> PSE | .22 | .23 | .11 | .35 |
| SI -> PSU | .25 | .26 | .14 | .38 |
| SI -> PE | .74 | .73 | .63 | .82 |
| SI -> RD | .60 | .61 | .51 | .70 |
| SI -> SU | .13 | .16 | .10 | .22 |
| UA -> AT | .04 | .07 | .03 | .15 |
| UA -> CO | .04 | .07 | .01 | .18 |
| UA -> CP | .06 | .09 | .04 | .17 |
| UA -> DC | .18 | .19 | .09 | .29 |
| UA -> EE | .13 | .13 | .04 | .26 |
| UA -> ER | .69 | .69 | .57 | .80 |
| UA -> FC | .05 | .08 | .04 | .15 |
| UA -> IU | .08 | .10 | .03 | .20 |
| UA -> PSE | .25 | .25 | .12 | .39 |
| UA -> PSU | .06 | .10 | .04 | .19 |
| UA -> PE | .03 | .08 | .03 | .16 |
| UA -> RD | .14 | .16 | .07 | .28 |
| UA -> SU | .59 | .59 | .47 | .70 |
| UA -> SI | .15 | .18 | .11 | .27 |

1. ^a^Neither confidence interval contains the value 1
2. ^b^AT = Attitude, CP = Compatibility, DC = Data Collection, ER = Errors, EE = Effort Expectancy, FC = Facilitating Conditions, IU = Intention to Use, PE = Performance Expectancy, PSE = Perceived Severity, PSU = Perceived Susceptibility, RD = Results Demonstrability, SI = Social Influence, SU = Secondary Use, UA = Unauthorised Access
